# Sustainability in the Interventional Radiology Suite: Environmental and Financial Implications

**DOI:** 10.1007/s00270-025-04185-6

**Published:** 2025-09-08

**Authors:** Patrick Cooper, Gráinne Allen, Réiltín Hayden, Iain Irvine, Ara Francis, Anthony G. Ryan

**Affiliations:** 1 Royal College of Surgeons in Ireland, Dublin, Ireland; 2 Division of Interventional Radiology, Department of Radiology, University Hospital Waterford, Waterford City, Ireland

**Keywords:** Sustainability, Climate change, Energy, Environment, Contrast medium, Materials, Cost, Financial

## Abstract

Healthcare waste accounts for a meaningful proportion of the global carbon footprint. There are innumerable global endeavours to integrate “green” initiatives into everyday living. Every interventional radiology (IR) department must strive to minimise its carbon footprint without any diminution of patient care. We explored the factors that negatively impact the environment with respect to waste generation in the IR suite and how simple innovations can bring about sustainable change. The aim of this review article is to outline the nature of waste generated in the IR suite and to describe changes that IR departments can make to reduce the negative environmental effect of their practice.

## Background

The United Nations has characterised climate change as the defining challenge of our era. Globally, countless initiatives aim to incorporate "green" practices into daily life, promoting sustainability across various sectors. Healthcare waste significantly contributes to the global carbon footprint, making it a critical area for environmental improvement. Within this context, it is of paramount importance for the IR department to actively work towards minimising its carbon footprint. This must be done without compromising the quality of patient care, ensuring that environmental responsibility goes hand-in-hand with maintaining the highest standards of medical treatment and without incurring additional costs. By implementing sustainable practices, the IR department can contribute to global efforts in reducing environmental impact while continuing to deliver excellent patient outcomes.

## Challenges and Solutions

Sustainability within the IR suite is often seen as a “side-project ” and approached as a grassroots effort rather than as a fully integrated institutional initiative. This means that sustainability measures are usually driven by individual or departmental efforts rather than being a central part of an institution’s goals. This attitude and perception has been challenged by Clements et al. who opined that, to be effective, these initiatives should be “top-down governance challenges”[1].

Departments frequently require financial incentives to motivate the reduction of their carbon footprint, yet such funding is often not made available. This lack of financial support can significantly hinder the implementation of effective sustainability practices. There are many factors that influence the effect of the IR suite on the environment. We explore some of the factors in this paper and outline changes that can be implemented on a local level. Some of these changes are minor and have no impact on procedure performance or the running of a list, but have a major effect on the environmental impact of an IR department.

## Excess Waste Generation

### Problems

Excess waste generation within the IR suite is a significant contributory factor impacting healthcare sustainability (Table 1). The suite typically produces large volumes of disposable materials, including single-use instruments and packaging. These materials are essential for maintaining sterility and ensuring patient safety, but their excessive use results in a substantial environmental footprint. One key issue contributing to this waste is over-preparation for procedures, where items are often opened in excess, leading to unneeded waste when items are not used but cannot be re-sterilised or reused.

**Table 1:** Excess waste generation in IR: problems and solutions

Problems	Solutions
Pre-emptive opening of supplies leads to unnecessary waste	Practice Just-in-Time Inventory—Open only the supplies and equipment immediately needed to minimise waste
Improper use of PPE (e.g. gloves, masks) when not clinically required	Education and Policy Enforcement—Train staff on appropriate PPE use to prevent overuse
Misuse of waste bin categories (e.g. recyclables mixed with general waste)	Implement Effective Waste Segregation Programs—Promote proper segregation to optimise disposal
Inefficient IR suite layout leading to poor waste segregation	Optimise IR Suite Layout—Position bins for recyclables, general waste, and hazardous materials in accessible locations
Lack of staff awareness on waste management	Clear Signage and Guidance—Provide clear instructions to ensure compliance with waste disposal protocols

Another challenge is the inappropriate use of personal protective equipment (PPE). While PPE is crucial for patient and healthcare worker safety, there are instances where its use is not clinically indicated, leading to unnecessary waste. For example, gloves, masks, and gowns may be used excessively or unnecessarily during procedures by members of staff not directly exposed to hazardous biomaterials, further increasing the volume of waste generated.

A third issue involves the improper disposal of materials due to incorrect waste bin categorisation. Even though recycling bins may be available, staff members may dispose of recyclable materials in general waste bins, hindering recycling efforts and exacerbating the overall waste problem. Additionally, the layout of the IR suite itself can contribute to inefficiencies in waste management, particularly when appropriate waste bins are not clearly labelled and are not strategically placed within easy reach, prompting staff to dispose of waste in the nearest bin regardless of its designated purpose.

### Solutions

Several approaches can be adopted to reduce excess waste generation in the IR suite [2, 3]. First, a *Just-in-Time inventory system* should be implemented. This approach advocates for opening only the necessary supplies and equipment for each procedure, rather than preemptively opening all items that *might* be required. This practice can significantly reduce the number of opened but unused items that ultimately contribute to waste.

Secondly, effective waste segregation programs are essential. Evidence suggests that comprehensive waste segregation in operating theatres can drastically reduce the amount of clinical waste sent for disposal. This approach not only benefits the environment but can also result in cost savings for healthcare institutions by optimising waste disposal processes and increasing recycling rates. Another solution is to introduce cost-effective recycling opportunities. By ensuring that recyclable materials are properly separated from general waste, hospitals can take advantage of recycling programs, which can offset disposal costs and reduce the overall environmental impact of waste. With respect to the large printed manuals included in many interventional radiology equipment boxes, industry partners could be discouraged from including them in every kit. A more sustainable approach would be to provide a QR code or similar digital link on the packaging to enable immediate online access to the relevant instructions when required.

Moreover, it is vital to optimise the layout of the IR suite to facilitate efficient waste segregation. Brassil et al. found that upon enquiry, many staff members believed that the general waste bin was actually for recyclable waste, as there was no recycle bin available in the IR suite [4]. Strategically placing clearly labelled bins for different types of waste—such as recyclables, hazardous waste, and general waste—ensures that staff can easily access the appropriate bins. This encourages proper waste disposal and improves overall waste management practices.

Finally, providing clear signage and guidance within the suite can help staff make informed decisions about waste disposal. Clear instructions on where and how to dispose of various types of waste will ensure that waste segregation is correctly implemented, thus enhancing compliance and reducing errors. By integrating these strategies into everyday operations, healthcare facilities can significantly reduce waste generation and move towards more sustainable practices without compromising patient care.

## Wastage of Iodinated Contrast Medium

### Problems

The wastage of iodinated contrast medium (CM) agents poses both environmental and economic challenges, some of which are referred to in Table 2. These agents are essential in many imaging procedures, but their overuse and improper disposal can lead to unnecessary waste and environmental contamination.

**Table 2:** Iodinated contrast medium waste: problems and solutions

Problems	Solutions
Inefficient use of contrast medium—Unused contrast medium is often discarded after procedures, leading to both environmental and financial waste	Considerations for Reusing Contrast Medium—Promote a conscientious approach to assess if residual contrast medium can be safely reused, reducing both waste and costs
Environmental contamination—Excess contrast medium excreted by patients can pollute water sources	Minimise Waste of Contrast Medium—Encourage assessment of whether urine collection and iodine recovery with a view to incineration is possible

Strategies such as dose reduction, urine collection, and AI-driven optimisation can help minimise environmental impact while maintaining imaging quality. In addition to environmental concerns, inefficiency in the use of CM represents a significant economic issue for healthcare providers. Often, after diagnostic or therapeutic procedures, the unused volume of CM is discarded, despite the possibility that residual CM could be used in subsequent procedures. This not only leads to the waste of valuable medical resources and environmental contamination as above, but also increases healthcare costs. This financial impact is discussed in greater detail below.

### Solutions

To address these issues, there are several key strategies that healthcare institutions can adopt. First, reducing the waste of CM requires minimising the volume of CM used per procedure*.* Healthcare professionals should be trained to accurately calculate and administer the minimum required amount of iodinated CM, ensuring that waste is minimised without compromising diagnostic quality. By adopting a more measured approach to CM usage, healthcare facilities can significantly reduce both the environmental and financial impacts of CM waste.

Second, healthcare providers should consider the reuse of residual CM whenever safe and feasible. In some cases, the residual CM in syringes or vials could be used for subsequent patients, provided it meets safety and sterility requirements. Encouraging clinicians to evaluate whether CM can be safely utilised for other procedures can help to reduce waste and conserve valuable medical resources [5]. This practice, while requiring careful assessment, aligns with principles of responsible resource stewardship and sustainability.

Finally, improving the management of iodinated CM waste is critical. One of the most pressing issues is the excretion of iodinated CM by patients in their urine, which, in large volumes, can contribute to significant contamination of water sources. For example, an estimated 71 tonnes of iodinated CM, primarily excreted by patients, contaminates the Rhine River annually [6]. This environmental burden underscores the urgent need for more sustainable management practices regarding CM. Recycling programmes provided by companies such as Bayer AG and GE HealthCare allow for the collection and recovery of iodine from residual CM, extending iodine’s lifecycle and reducing environmental impact [7]. Additionally, research suggests that urine collection systems could help capture a significant portion of excreted CM before it enters wastewater, with pilot studies showing a 45% reduction in CM concentration [6]. While the recovery of iodine from wastewater is currently not cost-effective, targeted collection prior to patient discharge and proper disposal strategies, such as incineration, could improve sustainability. Implementing these solutions at scale requires financial investment and logistical planning but could contribute to resource security while mitigating the environmental footprint of CM use. These principles can be applied to interventional radiology (IR), where high usage of CM underscores the need for improved recycling, waste reduction, and sustainable disposal strategies.

By adopting these solutions, healthcare facilities can effectively reduce iodinated CM waste, mitigate its environmental impact, and improve overall sustainability in medical imaging practices.

## Energy Wastage

Small, seemingly insignificant, changes can collectively have a profound impact on the sustainability of healthcare operations (Table 3), a good example of which is wise mindful energy management. Ensuring that lights, computers, and other electronic devices are turned off when not in use can greatly reduce unnecessary energy consumption, which, in turn, helps lower a healthcare facility’s carbon footprint. A study by Chua et al. found that equipment remaining on when not being used was a major cause for emissions in their centre and should be a main target for change in other centres [8].

**Table 3:** Sustainable actions and their impact

Action	Impact
Energy Management	Turning off lights and computers when not in use conserves energy and reduces operational costs
Minimising Packaging Waste	Opening packaging only when absolutely necessary reduces waste and its environmental footprint
Optimising Contrast Medium Usage	Administering the minimum effective dose prevents excess contrast medium waste while ensuring optimal patient care
Recycling Residual Contrast Medium	Adopting recycling practices reclaims iodine from residual contrast medium, promoting sustainability
Using Digital Documentation	Reducing reliance on printed materials lowers paper consumption and medical waste
Sustainable Procurement	Sourcing eco-friendly or biodegradable medical supplies minimises long-term environmental impact
Encouraging Reusable Equipment	Where safe and feasible, replacing single-use items with sterilised reusable alternatives reduces waste
Reducing Unnecessary Scans	Avoiding redundant imaging lowers radiation exposure and conserves resources such as contrast medium and electricity
Educating Staff on Sustainability	Raising awareness and training staff on eco-friendly practices fosters a long-term culture of environmental responsibility

This simple practice not only conserves energy but also contributes to the reduction of operating costs for medical institutions. The energy savings from such actions, although seemingly small on an individual scale, can add up to a significant impact when applied consistently across a healthcare setting. Furthermore, a study presented at the 2024 annual meeting of the Radiological Society of North America 2024 by Taskin et al. emphasised the importance of managing the energy usage of imaging systems, particularly angiography systems, by shutting them down during non-use periods [9]. The research suggests that these practices can result in substantial reductions in energy consumption, contributing to a significant reduction in the environmental impact of IR suites. This proactive approach to energy management is an essential step towards promoting sustainability in healthcare environments.

## Artificial Intelligence and Sustainability

Artificial intelligence (AI) in radiology presents a dual challenge: while its development and deployment are energy-intensive and contribute significantly to greenhouse gas emissions, it also offers opportunities to enhance environmental sustainability. Doo et al. aptly refer to AI as a double-edged sword with regards to its potential impact on sustainability [10]. Addressing the environmental impact of AI in radiology requires targeted strategies to minimise emissions while using its benefits for sustainability. AI can improve efficiency by shortening MRI scan times, optimising scanner scheduling, and reducing low-value imaging through decision support tools. These principles can be extrapolated to interventional radiology (IR), where AI-driven workflow optimisation, automated procedural planning, and intra-procedural image reconstruction techniques have the potential to reduce energy consumption and procedural waste. By integrating AI into IR practice, fluoroscopy times could be minimised, scheduling inefficiencies reduced, and resource use optimised, contributing to both environmental sustainability and improved patient outcomes.

## Financial Impact of Sustainable Practices

Adopting sustainable practices in IR has substantial financial implications that can contribute to both cost savings and operational efficiency. The initial investment in green technologies or practices can often be offset by the long-term savings that these initiatives generate. As healthcare institutions are continuously under pressure to reduce operating costs, sustainability efforts can serve as both a cost saving measure and a means of improving financial performance in the long run.

IR imaging systems consume significant amounts of energy even when not actively in use, leading to unnecessary electricity costs. A study measuring real-time energy consumption in multiple IR and cardiology suites found that systems remained in an idle state for extended periods, accounting for over 90% of total energy usage. By implementing simple operational adjustments—such as powering down systems overnight and on weekends—healthcare facilities could achieve substantial cost savings. The study estimated potential electricity cost savings of US$37,896 annually per hospital through such measures [11]. Beyond direct financial benefits, these savings could be reallocated towards patient care, equipment upgrades, or sustainability initiatives, making energy efficiency a financially prudent and environmentally responsible strategy.

Waste reduction is another critical financial consideration in IR. In a field that relies on consumables such as iodinated CM and single-use devices, optimising usage and reducing waste can lead to substantial financial savings. By carefully managing inventory and minimising waste—such as by recycling or reusing CM where possible—hospitals can lower their costs associated with purchasing new materials and managing waste disposal. When negotiating contracts for CM provision, hospital purchasing departments can be encouraged to favour those providers that will help the hospital develop and provide these facilities.

Moreover, the financial benefits of sustainability go beyond direct cost savings. Hospitals and healthcare facilities that adopt eco-friendly practices are increasingly viewed favourably by the public, which can enhance their reputation and patient trust. Many patients today are aware of environmental issues and increasingly prefer to seek care from institutions that prioritise sustainability. A recent study by Scholz et al. found that 66.2% of patients expressed willingness to adopt cost saving measures such as shorter hospital stays and increased follow-up visits, which could also contribute to lower healthcare expenditures [12]. These findings highlight the need for cost-neutral or cost saving sustainability strategies in healthcare to ensure widespread acceptance. As such, we must extrapolate patients’ willingness to “buy in” to sustainable healthcare and implement sustainable practices in IR. This may ultimately contribute to a department’s overall reputation, as well as helping to reduce its CO_2_ emissions. Additionally, healthcare providers who proactively embrace sustainability are better positioned to navigate potential regulatory changes, avoiding fines or penalties that could arise from non-compliance with environmental standards. The authors acknowledge that implementing some of these changes will be challenging in countries where hospitals and departments are financially incentivised to admit patients for procedures which could otherwise be performed as day cases.

In addition to cost savings, sustainable practices can also create opportunities for collaboration with industry partners and government bodies. Institutions that engage in green initiatives may be eligible for financial incentives or grants aimed at promoting sustainability in healthcare. These financial incentives can further support the implementation of sustainable technologies and practices, making them even more financially viable for healthcare facilities.

In summary, the financial case for sustainability in IR is compelling. By adopting energy-efficient technologies, reducing waste, and embracing eco-friendly practices, IR departments can reduce operational costs, improve efficiency, and enhance their financial and reputational standing. These financial benefits, coupled with the positive environmental impact, make sustainability not only an ethical choice but a practical and financially advantageous one for healthcare providers.

## Discussion

To encourage the development of sound environmental practices within their unit, IRs need to champion environmental sustainability in their daily conversations and professional practices. This means not only raising awareness of the environmental impact of interventional practice but also taking concrete actions in the clinical setting by putting the solutions described above into practice and using these as a springboard for developing newer, more effective, resource management solutions. Sustainable practices should be embedded in institutional policies, supported by staff training, and continuously evaluated for their effectiveness.

Furthermore, IRs have a unique opportunity to take a leadership role in promoting sustainability within the broader healthcare landscape. This entails advocating for institutional support for green initiatives, collaborating with manufacturers to develop more sustainable technologies (favouring those that do), and working together with other healthcare professionals to identify and address environmental challenges in clinical practice. By forming alliances with industry partners, interventional radiologists can influence the development of eco-friendly imaging technologies and materials, ensuring that sustainability becomes a core consideration in product design and implementation. Through these steps and future innovations, the environmental footprint of IR can be drastically reduced.

## Conclusion

Incorporating sustainability into medical practice is not just an ethical obligation, but also an opportunity for healthcare providers to improve patient care while safeguarding the environment. By embracing environmental responsibility as an integral part of their practice, interventional radiologists can make significant contributions towards achieving global sustainability goals. Through proactive engagement, innovation, and collaboration, IRs can play a pivotal role in the transformation of healthcare practices to meet the challenges of our time.

We have described small simple changes that IRs can effect that can collectively contribute to creating more environmentally responsible healthcare facilities. These changes, while modest in isolation, have the potential to foster a culture of environmental responsibility within the healthcare sector, ultimately benefitting both the planet and patient care. As healthcare professionals, it behoves us to apply the same principle of “First, do no harm” to the environment as we do in patient care.
